# Mesenchymal Stem Cell-derived Extracellular Vesicles Transmitting MicroRNA-34a-5p Suppress Tumorigenesis of Colorectal Cancer Through c-MYC/DNMT3a/PTEN Axis

**DOI:** 10.1007/s12035-021-02431-9

**Published:** 2021-10-08

**Authors:** Jiangning Zhao, Huanrong Lin, Kunsong Huang

**Affiliations:** 1 Gastrointestinal Peritoneal Cancer Surgery, The Fourth Clinical Medical School of Guangzhou University of Chinese Medicine, 1 Fuhua Road, Futian District, Shenzhen, 518033 Guangdong China; 2 Shenzhen Traditional Chinese Medicine Hospital, 1 Fuhua Road, Futian District, Shenzhen, 518033 Guangdong China; 3 Department of General Surgery, Guangzhou Overseas Chinese Hospital, The First Affiliated Hospital of Jinan University, Guangzhou, Guangdong China

**Keywords:** Colorectal cancer, Mesenchymal stem cells, Extracellular vesicles, MicroRNA-34a-5p, c-MYC, DNA methyltransferase 3a, Phosphatase and tensin homolog deleted on chromosome 10

## Abstract

Mesenchymal stem cell–derived extracellular vesicles (MSC-EV) can transport microRNAs (miRNAs) into colorectal cancer (CRC) cells, thus to inhibit the malignant phenotype of cancer cells. Whether MSC-EV could deliver miR-34a-5p to suppress CRC development was surveyed through the research. miR-34a-5p, c-MYC, DNA methyltransferase 3a (DNMT3a), and phosphatase and tensin homolog deleted on chromosome 10 (PTEN) expression were measured in CRC tissues and cell lines. miR-34a-5p and c-MYC expression were altered by transfection in HCT-116 cells. MSC-EV were transfected with miR-34a-5p- and c-MYC-related oligonucleotides and co-cultured with HCT-116 cells. HCT-116 cell growth after treatment was observed. Furthermore, the functional roles of miR-34a-5p and c-MYC were explored *in vivo*. The combined interactions of miR-34a-5p/c-MYC/DNMT3a/PTEN axis were assessed. miR-34a-5p and PTEN were downregulated while c-MYC and DNMT3a were upregulated in CRC. Depletion of miR-34a-5p drove while that of c-MYC restricted CRC cell growth. MSC-EV retarded CRC progression. Moreover, MSC-EV carrying overexpressed miR-34a-5p or depleted c-MYC further disrupted CRC cell progression. miR-34a-5p targeted c-MYC to regulate DNMT3a and PTEN. c-MYC overexpression abrogated EV-derived miR-34a-5p upregulation-induced effects on CRC. Restoring miR-34a-5p or depleting c-MYC in MSC-EV limited CRC tumor formation. MSC-EV-derived miR-34a-5p depresses CRC development through modulating the binding of c-MYC to DNMT3a and epigenetically regulating PTEN.

**Supplementary Information:**

The online version contains supplementary material available at 10.1007/s12035-021-02431-9.

## Introduction

Colorectal cancer (CRC) is a heterogeneous tumor presenting non-uniform molecular patterns that is stemmed from non-malignant adenomas [1]. Red and processed meat intake, alcohol abuse, obesity, inflammatory bowel disease, and family history of CRC consist of the risk factors for CRC [2]. CRC has atypical clinical symptoms in its early stages, leading to delayed diagnosis and treatment [3]. Clinically, since the 5-year survival rate of metastatic CRC is much more lower than that of CRC in stage I, promising targeted drug therapies have been developed for metastatic disease [4]. Moreover, omics technologies including metabolomics, transcriptomics, gnomics, and proteomics are utilized to improve diagnosis and treatment of CRC [5]. Thus, developments in targeted agents and armamentarium are of great importance to improve survival of CRC.

Extracellular vesicles (EV) are cell-derived membranous structures including exosomes and microvesicles [6]. Mesenchymal stem cell–derived exosomes are therapeutic tool in regenerative medicine [7] and develop an emerging strategy for CRC therapy due to their actions in growth and metastasis of cancer cells [8]. Secreted by tumor cells, exosomes contain various biomolecules that may induce angiogenesis [9] and CRC-derived exosomes have promising therapeutic value for CRC diagnosis [10]. Pharmacological approaches, such as drug encapsulation with exosomes are assumed to improve CRC treatment [11]. MSC-derived exosomes encapsulation has been once practiced with doxorubicin that offers a versatile platform to fight against CRC [12]. It is noted that bone marrow MSC-derived exosomes can deliver microRNAs (miRNAs) to restrict the tumorigenicity of CRC [13]. Dysregulated miR-34a-5p has been mentioned in CRC physiopathology [14]. miR-34a-5p has predictive value for recurrence and metastasis of CRC patients in stage II/III [15]. In fact, CpG-methylation of miR-34a is the mediated actor in primary CRC, as to cell drug resistance, and the processes of epithelial-mesenchymal transition (EMT) and metastasis [16]. Having ability to interact with miR-34a-5p [17], c-MYC is a proto-oncogene whose therapeutic inhibitor has been discovered to protect against CRC [18]. c-MYC stability is essential to regulate colon cancer (CC) cell survival [19], as well as invasive phenotype of CRC tumors [20]. c-MYC collaborates physically with DNA methyltransferase 3a (DNMT3a) [21] that is overexpressed in para-carcinoma tissues of sporadic CRC [22]. It is worthy that low doses of DNMT inhibitors can block the development of colitis-associated cancer [23]. DNMT3a can mediate epigenetic silencing of phosphatase and tensin homolog deleted on chromosome 10 (PTEN) [24] which is a mediator for the biological functions of CC cells [25, 26]. Based on those reports, this study aimed at elucidating the potential mechanism of miR-34a-5p/c-MYC/DNMT3a/PTEN axis in CRC.

## Methods and Materials

### Ethics Statement

The research protocol was approved by the ethics committee of The Fourth Clinical Medical School of Guangzhou University of Chinese Medicine; Shenzhen Traditional Chinese Medicine Hospital, and written informed consent was obtained. All animal experiments were conducted with the guidelines for the care and use of laboratory animals of the National Institutes of Health.

### Samples

CRC tissue and para-cancer normal tissue samples were obtained from The Fourth Clinical Medical School of Guangzhou University of Chinese Medicine; Shenzhen Traditional Chinese Medicine Hospital. All the 85 pairs of tissues were obtained through surgery, frozen, and stored in liquid nitrogen [27]. The clinicopathological characteristics of the patients were shown in Table 1.

**Table 1:** Clinicopathological characteristics of CRC patients

Clinicopathological characteristics	Cases (*n* = 85)	Percent (%)
Age (years)		
≤ 60	45	52.90%
> 60	40	47.10%
Gender		
Male	47	55.30%
Female	38	44.70%
Tumor stage		
I–II	36	42.40%
III	49	57.60%
Hereditary cancer		
Yes	21	24.70%
No	64	75.30%
Genetic screening		
Yes	32	37.60%
No	53	62.40%

### Cell Culture and Transfection

CRC cell lines HCT-116, SW-480, LoVo, and normal cell line HEK293 were acquired from ATCC (Rockville, MD, USA). Leibovitz’s L-15 medium was adopted for SW-480 cell culture, Ham’s F12K medium for HCT-116 and LoVo cell culture, and Dulbecco’s modified eagle medium (DMEM) for HEK293 cell culture. All the culture medium contained 10% fetal bovine serum (FBS).

miR-34a-5p- and c-MYC-related oligonucleotides sequences were provided by Sangon (Shanghai, China). The oligonucleotides were transfected into HCT-116 cells using Lipofectamine 3000 (Invitrogen, CA, USA), including negative control (NC)-inhibitor, miR-34a-5p-inhibitor, si-c-MYC and si-NC. Phosphate-buffered saline (PBS) was utilized as a NC [28].

### Extraction of EV

Bone marrow was mixed with MesenPRO RS medium (12746-012, Gibco, CA, USA) and separated using h-BM-MSC Isolation Kit (TBD). The collected cells were cultured with the medium changed every 2–3 days. Confluent monolayers of cells were observed 7 days later.

MSCs at 80% confluence were washed twice with PBS, and cultured in EV-free 10% FBS medium for 48 h. Then, the supernatant was centrifuged at 500 *g* for 15 min to remove cell debris, at 2000 *g* for 15 min to remove cell debris or apoptotic bodies, and at 10,000 *g* for 20 min to remove large vesicles. After filtering with a 0.22 micron filter, the sample was centrifuged at 110,000 *g* for 70 min at 4 °C, resuspended in PBS and ultra-centrifuged at 110,000 *g* for 70 min at 4 °C [29].

### Identification of EV

EV were fixed with 2% phosphotungstic acid at 4 °C, air-dried, observed under a TEM at 80 kV (FEI Tecnai G2 Spirit, Thermo Scientific, USA) and analyzed by western blot [30].

### Internalization of EV

EV and PKH26 (4 μL) were resuspended in Diluent C (1 mL), respectively. The EV suspension was mixed with the staining solution and added with an equal volume of 1% BSA. The labeled exosomes were ultracentrifuged at 100,000×*g* for 70 min and then ultracentrifuged again [31]. The PKH26-labeled EV were resuspended in 100 μL PBS.

HCT116 cells seeded on a 24-well plate at 1 × 10^6^ cells/well were routinely cultured for 12 h, and added with PKH26-labeled EV (10 μg/well) for 48-h reaction. The cells were fixed with 4% paraformaldehyde, stained with 4′,6-diamidino-2-phenylindole and observed under a fluorescence microscope. Red fluorescence represented the uptake of PKH26-labeled EV [32].

### Cells Co-culture with EV

HCT-116 and MSC were detached with trypsin, centrifuged at 1000 *g*, and then resuspended in DMEM (3 mL). Next, the suspension (1 mL) was diluted 20 times and counted. HCT-116 and MSC were spread over a co-culture chamber (0.4 mm) at 3:1. MSC (about 4 × 10^4^) were placed into the basolateral chamber containing 15% FBS-DMEM while HCT-116 cells (1 × 10^5^) in the root apical chamber with 10% FBS-DMEM. The medium was renewed every 1–2 days during the co-culture (4–5 days).

HCT-116 were co-cultured with MSCs which were transfected with NC-mimic, miR-34a-5p-mimic, si-NC, si-c-MYC, miR-34a-5p-mimic + oe-NC, and miR-34a-5p-mimic + oe-c-MYC [33].

### 3-(4, 5-dimethylthiazol-2-yl)-2, 5-diphenyltetrazolium bromide (MTT) assay

HCT-116 cells were seeded into 96-well plates. The culture medium (100 μL) was replaced with an equal volume of fresh medium containing 0.5 mg/mL MTT. After incubation of 0, 24, 48, and 72 h, 100 μL dimethyl sulfoxide (Sigma-Aldrich) was supplemented into cells and optical density_570 nm_ was measured by a microplate reader (Bio-Rad, Hercules, CA, USA) [27].

### Flow Cytometry

#### Apoptosis

HCT-116 cells were suspended in an Annexin V-fluorescein isothiocyanate (FITC) binding buffer, stained with Annexin V-FITC (Annexin V-FITC; Solarbio) and PI (Solarbio), and analyzed by a flow cytometer (BD Biosciences) [34].

### Transwell assay

Cell migration and invasion abilities were evaluated by transwell assay. Cells were suspended in serum-free medium and added into the upper cavity with diluted matrix gel (8-μm, BD Biosciences). The lower cavity contained 20% FBS. Cells after 48-h incubation were fixed with 4% paraformaldehyde, stained with 0.1% crystal violet and counted in five randomly-selected fields [35].

### Reverse Transcription Quantitative Polymerase Chain Reaction

Trizol reagent (Invitrogen) was employed to isolate total RNA from cells and tissues. mRNA and miRNA were reverse-transcribed into cDNA using the PrimerScript^TM^ RT Master Mix Kit and the PrimerScript miRNA RT-PCR kit and treated with qPCR with SYBR Green method on the CFX96 real-time PCR system (Bio-Rad). Glyceraldehyde-3-phosphate dehydrogenase (GAPDH) and U6 were internal controls. Table 2 listed all the primers. Data were collected through ABI 7500 real-time PCR machine (ABI, Foster City, CA, USA) and analyzed by the 2^−ΔΔCt^ method [36, 37].

**Table 2:** Primer sequences

Genes	Forward (5′-3′)	Tm	Reverse (5′-3′)	Tm
GAPDH	CATCCATGACAACTTTGGTATCGT	60.8	CATGAGGTCCAC	61.57
miR-34a-5p	TGGCAGTGTCTTAGCTGGTTGT	59.9	Uni-miR qPCR Primer, included in the kit	
DNMT3a	GCCCATTCGATCTGGTGATT	59.5	GGCGGTAGAACTCAAAGAAGAG	66
PTEN	ACACGACGGGAAGACAAGTT	56.8	TCCTCTGGTCCTGGTATGAAG	64
c-MYC	TCCGTCCTCGGATTCTCTGCTCT	66.3	GCCTCCAGCAGAAGGTGATCCA	60
E-cadherin	CAGCATCACTGGCCAAGGAGCTGA	70.4	GACCACACTGATGACTCCTGTGTTCC	59.1
Vimentin	CCGACACTCCT ACAAGATTTAGA	56.1	CAAAGATTTATTGAAGCAGAACC	58.84
N-cadherin	TTTGATGGAGGTCTCCTAACACC	60.0	ACGTTTAACACGTTGGAAATGTG	60
Snail	CCTCAAGATGCACATCCGAAGCCA	70.1	AGGAGAAGGGCTTCTCGCCAGTGT	62
U6	CTCGCTTCGGCAGCACA	59.3	AACGCTTCACGA	58

Note: GAPDH, glyceraldehyde-3-phosphate dehydrogenase; miR-34a-5p, microRNA-34a-5p; DNMT3a, DNA methyltransferase 3a; PTEN, phosphatase and tensin homolog deleted on chromosome 10

### Western Blot Assay

Total protein extracted from cells or tissues was lysed, separated by 6% sodium dodecyl sulfate-polyacrylamide gel electrophoresis, transferred to a polyvinylidene fluoride membrane, and blocked with 5% skimmed milk. The primary antibodies including c-MYC (1:1000, sc-40, Santa Cruz Biotechnology), DNMT3a (1:2000, NB120-13888, Novus Biologicals), PTEN (1:1000, 9559, Cell Signaling Technology), CD9 (1:1000, ab92726), CD63 (1:1000, ab59479), Calnexin (1:1000, ab22595, Abcam), HSP70 (1:1000, 4872, Cell Signaling Technology), and β-actin (1:1000, sc-47778, Santa Cruz Biotechnology), along with secondary antibody (7074, 1:2000, Cell Signaling Technology) were applied to incubate with the membrane. The intensity of the band was quantified by Image analysis system (Quantity One v4.62, Bio-Rad) [38].

### Dual Luciferase Reporter Gene Assay

The fragment of c-MYC 3′-UTR wild-type (WT) and mutant (MUT) containing miR-34a-5p-binding sites was cloned into pmirGLO (Promega, Madison, USA). HCT-116 cells were transfected with WT/MUT-c-MYC vector and miR-34a-5p-mimic/NC-mimic through Lipofectamine 3000 (Life Technologies Corporation, Carlsbad, CA, USA). Promega dual luciferase system (Glomax 20/20, ATCC) was utilized to measure firefly and Renilla luciferase activities, thus to determine relative luciferase activity [39].

### Co-immunoprecipitation Assay

Cell extracts were reacted with IgG and protein A/G agarose to eliminate non-specific binding, added with 2 μg DNMT3a antibody (1:2000, NB120-13888, Novus Biologicals) and centrifuged. Then, A/G agarose were suspended in 2× sodium dodecyl sulfate and heated at 100 °C. The immunoprecipitated protein was analyzed by 12% sodium dodecyl sulfate polyacrylamide gel electrophoresis, transferred to a polyvinylidene fluoride membrane, and blocked overnight. c-MYC (1:1000, sc-40, Santa Cruz Biotechnology) and PTEN (1:50, 9559, Cell Signaling Technology) were diluted and reacted with the membrane, followed by incubation with the secondary antibody. Finally, data analysis was performed by Odyssey infrared imaging system (LiCorBioSciences, Lincoln, NE, USA) [40].

### Tumor Xenografts in Nude Mice

Male BALB/c nude mice (4–5 weeks) were raised in an environment of specific pathogen-free grade. Mice were subcutaneously injected with HCT-116 cells (1 × 10^6^) co-cultured with transfected MSCs in the right abdomen. Mice for control were injected with PBS-treated HCT-116 cells. The average tumor diameter was recorded regularly every 4 days. After 24 days, the mice were euthanized and the tumor weight was recorded [41–43].

### Statistical Analysis

Statistical analysis was finished by SPSS 18.0 (SPSS, Chicago, USA). Analysis of variance was utilized to evaluate the statistical differences of groups. *P* < 0.05 was considered a significant difference [44].

## Results

### Identification of MSC and EV

**Fig. 1:**
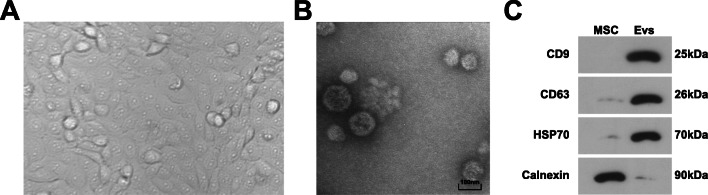
Identification of MSC and EV. **A** Observation of MSCs; **B** TEM observed MSC-EV; **C** Western blot tested surface antigens of MSC and EV (CD9, CD63, HSP70, and Calnexin)

MSC-derived exosomes serve as the inhibitor in pancreatic cancer [33]. MSC obtained from patients undergoing hip arthroplasty showed plastic adhesion, growing like fibroblast-like spindles after 2–3 passages (Fig. 1A).

Viewed by a TEM, the extracted EV from MSC were about 50–130 nm (Fig. 1B). Moreover, Western blot identified the surface antigens of EV, and discovered that, CD9, CD63, and HSP70 were positively expressed while Calnexin was negatively expressed (Fig. 1C).

### miR-34a-5p is Downregulated in CRC

**Fig. 2:**
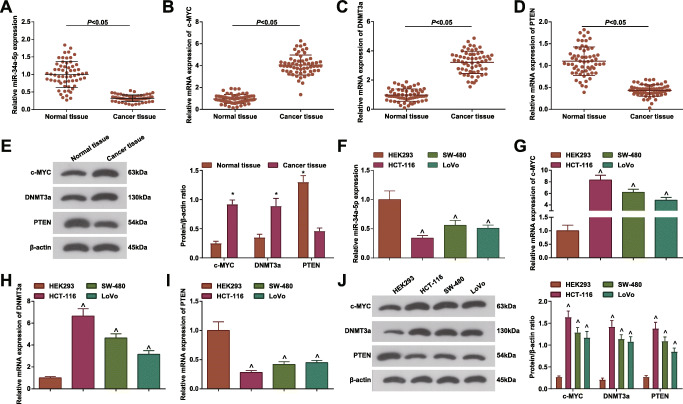
miR-34a-5p is down-regulated in CRC. **A**–**D** RT-qPCR tested miR-34a-5p, c-MYC, DNMT3a and PTEN mRNA expression in cancer tissues and normal tissues, cases = 60; **E** Western blot tested c-MYC, DNMT3a, and PTEN protein expression in cancer tissues and normal tissues; **F**–**I**. RT-qPCR tested miR-34a-5p, c-MYC, DNMT3a, and PTEN mRNA expression in HEK293, HCT-116, SW-480, and LoVo cells; **J** Western blot tested c-MYC, DNMT3a and PTEN protein expression in HEK293, HCT-116, SW-480 and LoVo cells; The data were expressed as mean ± standard deviation of three independent experiments. ^*^P < 0.05 compared with normal tissues; ^*P* < 0.05 compared with HEK293 cells

It has been reported that miR-34a-5p is downregulated in CRC [15] while c-MYC is involved in the regulation of CC, showing a high expression trend [45]. miRNA can affect the tumor microenvironment by affecting the level of protein. DNMT3a can regulate the development of CC, and generally shows a high expression trend in CC cells, and the regulation of DNMT3a may be related to the expression of miRNA [46]. At the same time, PTEN is lowly expressed in CC and is closely related to the development of CC [47]. RT-qPCR and Western blot tested the downregulated miR-34a-5p and PTEN, and upregulated c-MYC and DNMT3a in cancer tissues and cells (Fig. 2A–J). Among CRC cell lines, miR-34a-5p and PTEN showed the lowest expression while c-MYC and DNMT3a showed the highest levels in HCT-116 cells, so HCT-116 cells were adopted for in vitro experiments.

### Inhibiting miR-34a-5p Promotes CRC Cell Development

**Fig. 3:**
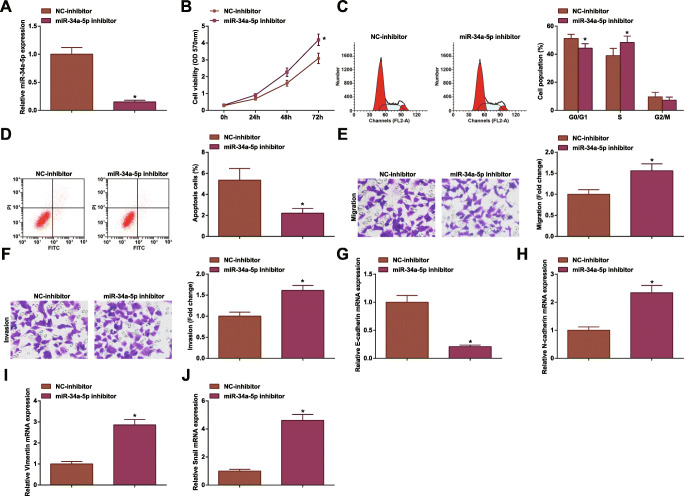
Inhibiting miR-34a-5p promotes CRC cell development. **A** RT-qPCR tested miR-34a-5p expression in HCT-116 cells after miR-34a-5p inhibition; **B** MTT tested viability of HCT-116 cells after miR-34a-5p inhibition; **C** flow cytometry tested apoptosis of HCT-116 cells after miR-34a-5p inhibition; **D** Transwell assay tested migration of HCT-116 cells after miR-34a-5p inhibition; **E** Transwell assay tested invasion of HCT-116 cells after miR-34a-5p inhibition; **F** RT-qPCR tested E-cadherin mRNA expression in HCT-116 cells after miR-34a-5p inhibition; **G** RT-qPCR tested N-cadherin mRNA expression in HCT-116 cells after miR-34a-5p inhibition; **H** RT-qPCR tested Vimentin mRNA expression in HCT-116 cells after miR-34a-5p inhibition; **I** RT-qPCR tested Snail mRNA expression in HCT-116 cells after miR-34a-5p inhibition; the data were expressed as mean ± standard deviation of three independent experiments. ^*^P < 0.05 compared with the NC-inhibitor group

The effect of miR-34a-5p on the occurrence and development of CRC were clarified by various experiments. After successful transfection with miR-34a-5p inhibitor (Fig. 3A), HCT-116 cell proliferation, migration and invasion were enhanced, and apoptosis was inhibited (Fig. 3B–E).

EMT contributes to the metastasis of cancer cells and is associated with more aggressive cancer phenotypes. EMT-related proteins in CC are related to tumor development [48]. EMT is characterized by reduced level of epithelial marker E-cadherin, accompanied by increased expression of mesenchymal markers Vimentin, N-cadherin, and Snail [49]. As measured, E-cadherin level was decreased while N-cadherin, Vimentin, and Snail levels were raised in HCT-116 cells after downregulating miR-34a-5p (Fig. 3F–I).

### MSC-EV Suppress CRC Progression

**Fig. 4:**
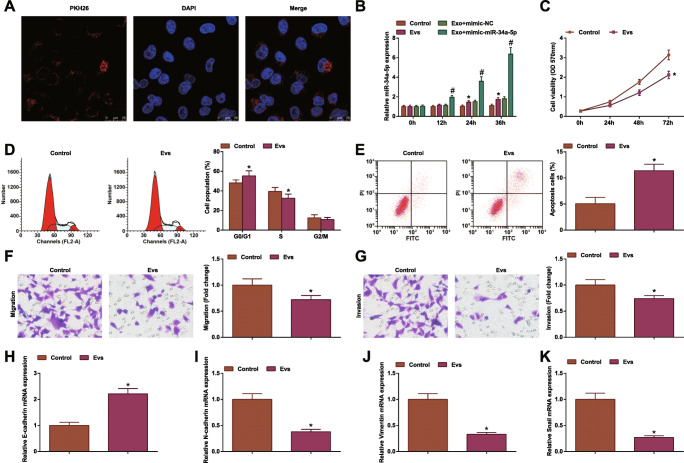
MSC-EV suppress CRC progression. **A** PKH26 staining observed uptake of EV; **B** RT-qPCR tested miR-34a-5p expression in HCT-116 cells after co-culture of EV; **C** MTT tested viability of HCT-116 cells after co-culture of EV; **D** flow cytometry tested apoptosis of HCT-116 cells after co-culture of EV; **E** Transwell assay tested migration of HCT-116 cells after co-culture of EV; **F** Transwell assay tested invasion of HCT-116 cells after co-culture of EV; **G** RT-qPCR tested E-cadherin mRNA expression in HCT-116 cells after co-culture of EV; **H** RT-qPCR tested N-cadherin mRNA expression in HCT-116 cells after co-culture of EV; **I** RT-qPCR tested Vimentin mRNA expression in HCT-116 cells after co-culture of EV; **J** RT-qPCR tested Snail mRNA expression in HCT-116 cells after co-culture of EV; the data were expressed as mean ± standard deviation of three independent experiments. ^*^P < 0.05 compared with the control group

MSC-EV mediate cancer development by changing tumor microenvironment [43]. Proved by PKH26 staining, HCT-116 cells having been co-cultured with MSC-EV smoothly internalized MSC-EV (Fig. 4A). Based on the theory that MSC-EV can transfer miRNAs to other cells [50], HCT-116 cells were co-cultivated with MSC-EV that had been transfected with miR-34a-5p-mimic. Then, the results displayed that miR-34a-5p expression was increased in HCT-116 cells in a time-dependent manner (Fig. 4B).

After co-culture with MSC-EV, HCT-116 cells were characterized by inhibited cell viability, migration and invasion, promoted apoptosis, augmented E-cadherin expression, and reduced Vimentin, N-cadherin, and Snail expression (Fig. 4C–J).

### Inhibiting c-MYC Hinders CRC Cell Development

**Fig. 5:**
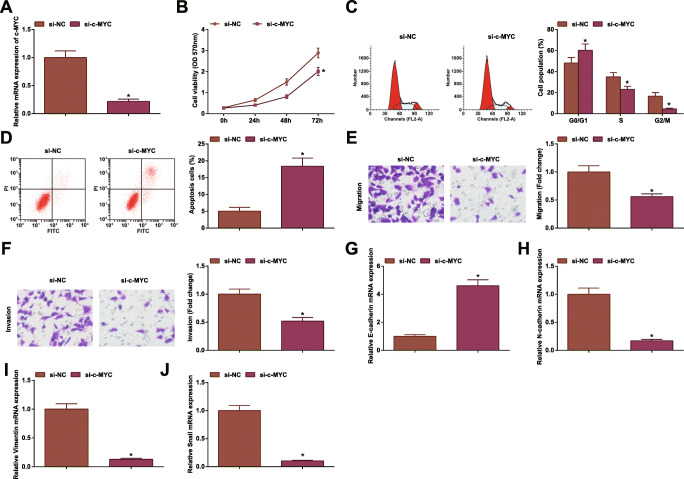
Inhibiting c-MYC hinders CRC cell development. **A** RT-qPCR tested c-MYC expression in HCT-116 cells after c-MYC inhibition; **B** MTT tested viability of HCT-116 cells after c-MYC inhibition; **C** flow cytometry tested apoptosis of HCT-116 cells after c-MYC inhibition; **D** Transwell assay tested migration of HCT-116 cells after c-MYC inhibition; **E** Transwell assay tested invasion of HCT-116 cells after c-MYC inhibition; **F** RT-qPCR tested E-cadherin mRNA expression in HCT-116 cells after c-MYC inhibition; **G** RT-qPCR tested N-cadherin mRNA expression in HCT-116 cells after c-MYC inhibition; **H** RT-qPCR tested Vimentin mRNA expression in HCT-116 cells after c-MYC inhibition; **I** RT-qPCR tested Snail mRNA expression in HCT-116 cells after c-MYC inhibition; the data were expressed as mean ± standard deviation of three independent experiments. ^*^P < 0.05 compared with the si-NC group

Our experiments in tissues and cells further confirmed upregulation trend of c-MYC in CRC. To delve the mechanism of c-MYC in CRC, si-c-MYC was successfully transfected into HCT-116 cells (Fig. 5A), after which HCT-116 cell progression and EMT were found to be retarded (Fig. 5B–I).

### MSC-EV Carrying Upregulated miR-34a-5p or Downregulated c-MYC Further Disrupt CRC Cell Progression

**Fig. 6:**
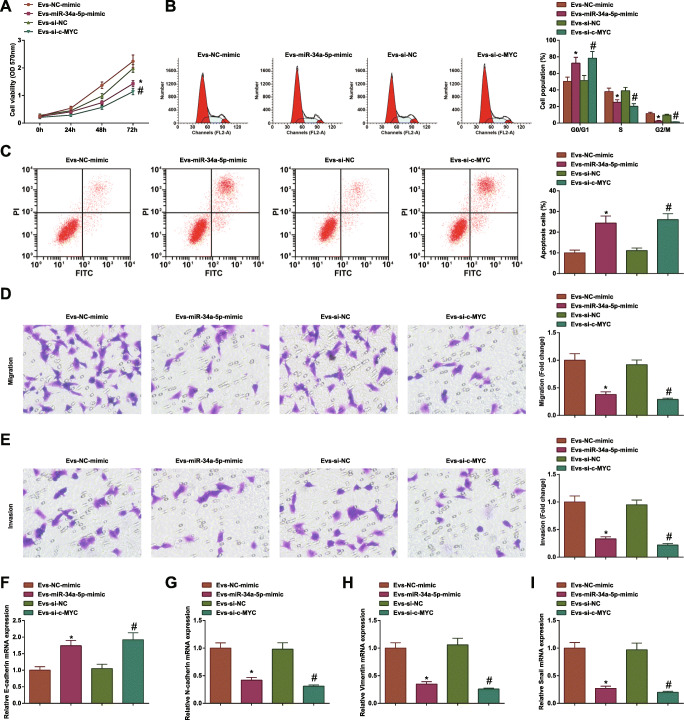
MSC-EV carrying upregulated miR-34a-5p or downregulated c-MYC further disrupt CRC cell progression. **A** MTT tested viability of HCT-116 cells after co-culture of EV; **B** flow cytometry tested apoptosis of HCT-116 cells after co-culture of EV; **C** Transwell assay tested migration of HCT-116 cells after co-culture of EV; **D** Transwell assay tested invasion of HCT-116 cells after co-culture of EV; **E** RT-qPCR tested E-cadherin mRNA expression in HCT-116 cells after co-culture of EV; **F** RT-qPCR tested N-cadherin mRNA expression in HCT-116 cells after co-culture of EV; **G** RT-qPCR tested Vimentin mRNA expression in HCT-116 cells after co-culture of EV; **H** RT-qPCR tested Snail mRNA expression in HCT-116 cells after co-culture of EV; the data were expressed as mean ± standard deviation of three independent experiments. ^*^P < 0.05 compared with the EV-NC-mimic group; ^#^*P* < 0.05 compared with the EV-si-NC group

EV from MSC transfected with miR-34a-5p mimic or si-c-MYC were co-cultured with CRC cells for further research. It was confirmed that MSC-EV-containing miR-34a-5p mimic or si-c-MYC further limited CRC cell growth and EMT process (Fig. 6A–H). The results informed that MSC-EV carrying upregulated miR-34a-5p or downregulated c-MYC further disrupted CRC cell progression.

### miR-34a-5p Targets c-MYC

**Fig. 7:**
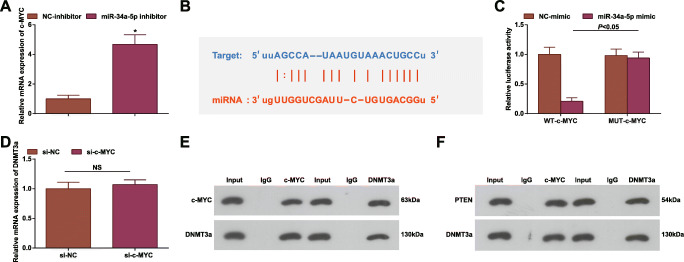
miR-34a-5p targets c-MYC. **A** RT-qPCR tested c-MYC expression in HCT-116 cells after miR-34a-5p inhibition; **B** Bioinformatics website Starbase predicted the binding site of miR-34a-5p and c-MYC; **C** luciferase reporter gene experiment verified the targeting relationship between miR-34a-5p and c-MYC; **D** RT-qPCR tested DNMT3a mRNA expression in HCT-116 cells after c-MYC inhibition; **E** CO-IP assay tested the interaction between c-MYC and DNMT3a; **F** CO-IP assay tested the interaction between PTEN and DNMT3a; the data were expressed as mean ± standard deviation of three independent experiments. ^*^P < 0.05 compared with the NC-inhibitor group

Though the regulatory functions of miR-34a-5p and c-MYC have been clarified in CRC, their combined action is still unknown. Given that, we surveyed whether c-MYC is involved in miR-34a-5p regulating CRC. Firstly, c-MYC level after transfection of miR-34a-5p inhibitor was measured and the finding manifested the incremental c-MYC level in cells (Fig. 7A). Later, the targeting relationship of miR-34a-5p and c-MYC was predicted on the Starbase website (Fig. 7B) and further confirmed by dual luciferase report experiment (Fig. 7C).

There is an interaction between c-MYC and DNMT3a [21]. In our study, their interaction was also validated. DNMT3a expression was reduced after inhibiting c-MYC (Fig. 7D). Moreover, two-way CO-IP assay mirrored that c-MYC and DNMT3a were co-precipitated in HCT-116 cells by anti-c-MYC antibody. Co-immunoprecipitation with anti-DNMT3a antibody further confirmed their interaction (Fig. 7E). PTEN has been mentioned as the mediated gene of DNMT3a [24]. Actually, CO-IP assay also validated their interaction (Fig. 7F).

### c-MYC Overexpression Abrogates EV-containing miR-34a-5p Upregulation-induced Effects on CRC

**Fig. 8:**
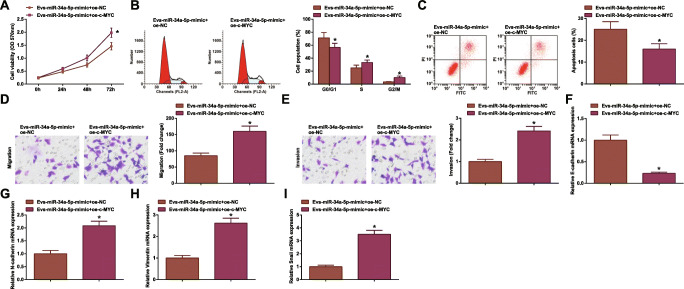
c-MYC overexpression abrogates EV-mediated miR-34a-5p upregulation-induced effects on CRC. **A** MTT tested viability of HCT-116 cells after co-culture of EV; **B** flow cytometry tested apoptosis of HCT-116 cells after co-culture of EV; **C** Transwell assay tested migration of HCT-116 cells after co-culture of EV; **D** Transwell assay tested invasion of HCT-116 cells after co-culture of EV; **E** RT-qPCR tested E-cadherin mRNA expression in HCT-116 cells after co-culture of EV; **F** RT-qPCR tested N-cadherin mRNA expression in HCT-116 cells after co-culture of EV; **G** RT-qPCR tested Vimentin mRNA expression in HCT-116 cells after co-culture of EV; **H** RT-qPCR tested Snail mRNA expression in HCT-116 cells after co-culture of EV; the data were expressed as mean ± standard deviation of three independent experiments. ^*^P < 0.05 compared with the EV-miR-34a-5p-mimic + oe-NC group

The combined effects of MSC-EV, miR-34a-5p, and c-MYC were further discussed in the field of CRC. Series of assays experimentally presented that though MSC-EV delivering miR-34a-5p-mimic depressed CRC cell growth and EMT process, another transfection of oe-c-MYC into MSC-EV would abrogate the therapeutic effects of miR-34a-5p-mimic (Fig. 8A-H).

### Restoring miR-34a-5p or Depleting c-MYC in MSC-EV Limits Tumor Formation in CRC

**Fig. 9:**
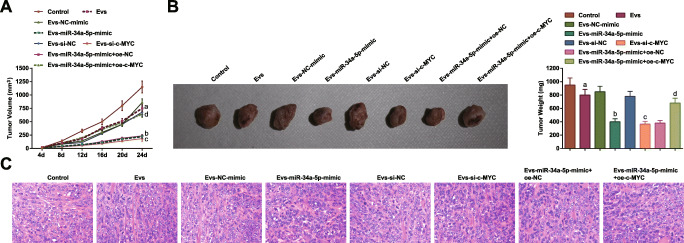
Restoring miR-34a-5p or depleting c-MYC in MSC-EV limits tumor formation in CRC. **A** Tumor volume of nude mice; **B** tumors and tumor weight of nude mice; **C** HE staining detected the histopathology of tumors; the data were expressed as mean ± standard deviation of three independent experiments. a *P* < 0.05 compared with the control group; b *P* < 0.05 compared with the EV-NC-mimic group; c *P* < 0.05 compared with the EV-si-NC group; d *P* < 0.05 compared with the EV-miR-34a-5p-mimic + oe-NC group

Tumor xenograft in nude mice was carried out to study whether miR-34a-5p/c-MYC/DNMT3a axis regulates CRC in vivo. HCT-116 cells co-cultured with MSC-EV were injected into mice (*n* = 5/group). The results revealed that MSC-EV reduced tumor volume and weight in mice, and MSC-EV transporting miR-34a-5p-mimic further enhanced the tumor-suppression effects. In addition, the anti-tumor effect of MSC-EV-derived miR-34a-5p was reversed by c-MYC overexpression (Fig. 9A, B).

Sections of tumors were analyzed by HE staining. It was displayed that the tumor tissues without any treatment were necrotic and accompanied by inflammatory cell infiltration, and the tumor cells were irregular in size and shape. The tumor tissues from mice injected with HCT-116 cells co-cultured with MSC-EV showed attenuated inflammatory cell infiltration. In response to upregulation of miR-34a-5p or downregulation of c-MYC on the basis of MSC-EV treatment, the degree of inflammatory cell infiltration in tissue sections was the lowest (Fig. 9C). It was indicated that miR-34a-5p/c-MYC/DNMT3a axis regulated PTEN to suppress CRC tumor growth in vivo.

## Discussion

CRC is the 3^rd^ prevalent malignancy, in which metastasis is the main cause of cancer-related deaths [51]. Our study has delineated the action of miR-34a-5p/c-MYC/DNMT3a/PTEN axis in CRC. At the beginning, we made efforts to discuss the role of miR-34a-5p in CRC and then discovered that miR-34a-5p was downregulated in the disease and its downregulation promoted on cellular growth and EMT process. Next, we confirmed c-MYC as the target of miR-34a-5p and knocking down c-MYC repressed CRC development and EMT. Subsequently, we also defined MSC-EV as a protector against CRC, while the combined function of MSC-EV with upregulated miR-34a-5p or downregulated c-MYC was better to control CRC in vitro and in vivo (Supplementary Figure S1).

miR-34a-5p has been marked with anti-tumor effects in cancers, including but not limited to CRC. In a latest report, suppressed miR-34a-5p showcases in CRC and long non-coding RNA-mediated miR-34a-5p downregulation is attributable to strengthen invasion and metastasis of CRC cells [52]. A preceding paper has mentioned the reduced level of miR-34a-5p in CRC, and increased miR-34a-5p level introduced to CRC cells would limit cell proliferation, migration, invasion, and metastasis, as well as induce cell apoptosis [15]. In the area of prostate cancer, miR-34a-5p expression is lower than the basic level and lncRNA-meidated knockdown of miR-34a-5p can elevate disheveled associated activator of morphogenesis 1, thus to accelerate cancer development [53]. As to liver cancer, miR-34a-5p exerts anti-proliferative, anti-migratory, and anti-invasive effects on cancer cells through targeted regulation of Yin and Yang 1 [54]. As for esophageal squamous cell carcinoma, cancer cells containing upregulated miR-34a-5p exhibit the impaired proliferation, migration, invasion, and EMT [55]. As to the combined functions of miRNA with EV, a published article has unraveled that miR-34a can be delivered by human bone marrow MSC-derived exosomes into glioblastoma cells, thereby disrupting cell proliferation, invasion, migration, and tumorigenesis [56]. To our surprise, MSC-derived exosomal miR-34c into nasopharyngeal carcinoma exerts to frustrate cell aggressiveness and EMT process [57]. Other than that, MSC-derived exosomes could convey miR-3940-5p overexpression vector to CRC cells, thus to obstruct EMT process, cell invasion, and tumor metastasis [58]. Besides, bone marrow MSC-derived exosomes functionally cooperate with miR-124 in pancreatic adenocarcinoma cells, so as to inhibit cell proliferation and EMT [59]. All of the reports support the positive role of miR-34a-5p in cancer prevention, as well as the collaborative effects of EV and miRNAs on cancers.

It has been fundamentally based that miR-34 can target c-Myc [60, 61]. In fact, similar to our finding in CRC, another report studying high-grade colon adenocarcinoma cells has examined the widespread c-MYC [62]. Pivoted on the actual role in cancer development, a study has once recorded that induction of c-Myc expression facilitates RING finger protein 8 to promote CC cell proliferation [63]. Oppositely, an inhibitor for c-MYC, compound 42 has been developed to fight against CRC cell proliferation and differentiation [18]. A reported article has mentioned that though miR-34b-5p upregulation represses colitis-associated cancer aggravation, c-MYC overexpression is able to abrogate the suppressed malignant phenotypes [64]. Mechanistically, miR-34b works as an inhibitor for CC cell proliferation but an inducer for cell apoptosis, in which c-MYC expression renders meritorious service [65]. For CC stem cells, deceased c-MYC expression in cells obstructs cell invasion and migration abilities [66]. In consistent with the papers, the pro-tumor actions of c-MYC are further validated in gastrointestinal cancers.

Determined in the present work, c-MYC could bind to DNMT3a, thus to regulate PTEN. Previously, a study report has decoded that MYC interacts with DNMT3a, thus silencing miR-200b and leading to EMT in triple-negative breast cancer [67]. DNMT3a is defined as one of the overexpressed genes in sporadic CRC [22] and theaflavin, an inhibitor for DNMT (DNMT1 and DNMT3a) can combat CC cell proliferation and progression [68]. It is interestingly identified that DNMT3a upregulation partially reduces PTEN level in hepatocellular carcinoma cells [69], showing the inverse relation between DNMT3a and PTEN. PTEN is an actor that strains CRC cell proliferation and migration [70] and PTEN upregulation could prevent proliferation and induce apoptosis of chemo-resistant CC cells [71]. All in all, the potential performances of DNMT3a and PTEN have been further studied in CRC.

## Conclusion

To conclude, miR-34a-5p is anti-tumor in CRC through targeting c-MYC to control DNMT3 and PTEN. It is a novel research to navigate the mechanisms underlying CRC pathology, and more experimental researches are still needed for better understanding the miR-34a-5p-related mechanism in cancers.

## Supplementary Information

Supplementary Figure 1 MSC-EV transmitting miR-34a-5p suppress tumorigenesis of CRC through c-MYC/DNMT3a/PTEN axis. (PNG 80 kb)

High resolution image (EPS 1375 kb)
