# Supplementary figures and images for: Mesenchymal Stem Cell-derived Extracellular Vesicles Transmitting MicroRNA-34a-5p Suppress Tumorigenesis of Colorectal Cancer Through c-MYC/DNMT3a/PTEN Axis
Source: Mol Neurobiol. 2021 Oct 8;59(1):47–60. doi: 10.1007/s12035-021-02431-9 (PMC8786758; doi:10.1007/s12035-021-02431-9)

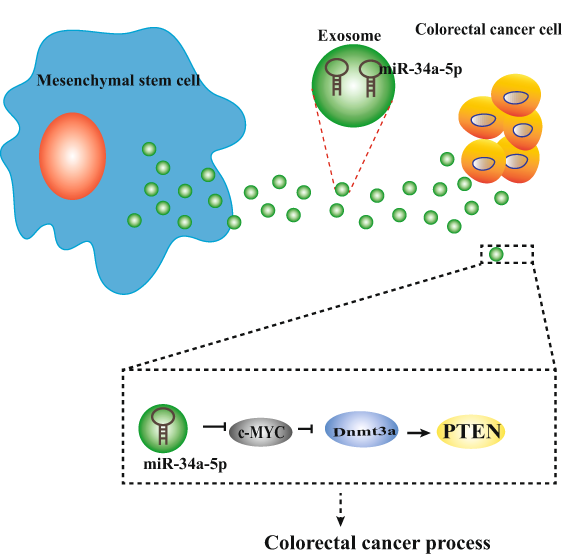

Supplement: Supplementary file 1 — MSC-EV transmitting miR-34a-5p suppress tumorigenesis of CRC through c-MYC/DNMT3a/PTEN axis. (PNG 80 kb) [file 12035_2021_2431_Fig10_ESM.png]
